# From environmental concerns to nationalist waves: Mapping the evolving Fukushima wastewater discourse on Weibo

**DOI:** 10.1371/journal.pone.0352452

**Published:** 2026-07-15

**Authors:** Siwei Qin, Wonjae Lee

**Affiliations:** Graduate School of Culture Technology, KAIST, Daejeon, Republic of Korea; University of Naples Federico II: Universita degli Studi di Napoli Federico II, ITALY

## Abstract

This study employed Structural Topic Modeling (STM) on 56,526 Weibo posts to examine the evolving discourses surrounding the Fukushima wastewater release. While rational discourse focusing on technical and environmental concerns initially coexisted and competed with nationalist narratives, the discourse rapidly transformed into predominantly nationalist rhetoric driven by grassroots users. Nationalism discourse was framed through two antagonisms: 1) direct, volatile anti-Japanese sentiment rooted in historical grievances and 2) broader, consistent anti-Western skepticism anchored in geopolitical rivalries. Moving beyond the binary paradigm of Chinese cyber-nationalism as either purely top-down mobilization or bottom-up explosion, this study reveals a nuanced interplay between actor types: grassroots users predominantly led nationalistic discourses, especially anti-Japanese discourse, while Key Opinion Leaders (KOLs) focused on technical discussions but occasionally amplified anti-Western sentiment. By identifying a latent nationalist sensitivity and a victimhood-centric view of international affairs, the study demonstrates how affective public sentiment can transform environmental issues into perceived insults to national dignity, intensified by platform-mediated grassroots agency.

## Introduction

In August 2023, Japan began discharging treated radioactive water from the Fukushima Daiichi Nuclear Power Plant into the Pacific Ocean, following approval by the International Atomic Energy Agency (IAEA). This decision sparked a surge of heated debate and nationalist sentiment on Chinese social media platforms, where environmental anxieties, historical grievances, and geopolitical tensions intersected.

The Fukushima wastewater controversy illustrates how social media reconfigures nationalism as a continuous process embedded in algorithmic infrastructures rather than an episodic elite discourse [1,2]. National identity is enacted through high-frequency micro-practices of moral condemnation and historical invocation, consistent with Durkheimian theories of collective emotion and interaction ritual, while departing from institution-centered models of nationalist reproduction [3–5]. Environmental risk operates less as a technical concern than as a moralized and secularized symbol enabling geopolitical antagonism without overt escalation [6,7]. Platform algorithms privilege emotionally charged, conflictual content, reinforcing us–them boundaries and blurring the line between public deliberation and international politics [8–11].

Nationalism is a multifaceted phenomenon encompassing civic pride, cultural identity, institutional loyalty, and ethno-symbolic resources [12]. In recent decades, the distinction between in-group and out-group entities has emerged as an increasingly noted analytical dimension in the era of digital communication. The growing popularity and infrastructural embeddedness of social media have made nationalism—especially in its more aggressive and exclusionary manifestations—increasingly visible in everyday public discourse [13]. This intensified public salience is observable across diverse contexts, from populist movements that frame international cooperation as threats to domestic sovereignty [14] to pandemic-era narratives that systematically attribute blame to foreign groups [15]. Anderson [1]’s notion of “imagined political communities” thus acquires a renewed, digitally mediated inflection: while print and broadcast media once served as primary vehicles for imagining the nation through differentiation, digital platforms now fulfill this role through algorithmically curated content that amplifies exclusionary nationalism’s expressive power and political utility.

This boundary-making logic of nationalism also holds particular significance in the Chinese context, where the historical narrative of the “Century of National Humiliation” (百年国耻) continues to shape collective memory, fueling anti-Japanese and anti-Western sentiments [16,17]. These sentiments are not merely historical relics; they are actively reproduced in contemporary contexts, from maritime disputes with Japan to the US-China trade war [18]. The outbreak of COVID-19 further amplified this exclusionary logic, with the dichotomy of the “innocent us” versus the “despicable others” becoming a central theme in official nationalist rhetoric [19]. Propaganda strategies of “us” and “them” dichotomy are both reinforced by state-controlled media and censorship [20] and internalized by the public, shaping the contours of nationalist discourse in the digital age.

Existing studies on Chinese nationalism have mainly relied on static methodological approaches that hardly capture the dynamic, event-driven nature of nationalist discourse. Ko [21] employed cross-sectional survey experiments to assess nationalist sentiment effects on foreign policy preferences, while Burcu [20] used discourse analysis of media content and interviews during discrete time windows of the Diaoyu/Senkaku crises. Although these approaches provide valuable insights, they cannot track how nationalist narratives emerge, evolve, and compete for attention as events unfold in real-time. This study addresses these temporal and analytical limitations by employing Structural Topic Modeling (STM) to systematically analyze the continuous evolution of nationalist discourse on Chinese social media throughout the Fukushima wastewater incident.

Existing scholarship has mainly anchored Chinese nationalism within zero-sum territorial disputes [22,23]. This study advances the literature by shifting focus to global environmental crises as a unique locus for nationalist mobilization. The Fukushima incident serves as a strategic case to test the boundaries of nationalism in this transboundary context, presenting a puzzle: how global ecological risks, despite aligning with Chinese official cosmopolitan rhetoric of ‘Community with a Shared Future for Mankind’ [24], are paradoxically reframed through the lens of historical grievance. By analyzing the tension between rationalism and nationalism, this study aims to elucidate the mechanisms through which the ‘other’ is constructed even without direct territorial encroachment.

Lastly, existing studies on Chinese online nationalism tend to examine different social actors in isolation. Zhang [25] assume that key opinion leaders (KOLs) on Weibo are the most influential actors, treating their voices as representative of the dominant political opinions in the online public sphere. Other scholars focus exclusively on the role of grassroots youth in generating nationalist content, termed as “fandom nationalism” [26]. However, these approaches often overlook the possibility that different social actors may hold divergent preferences and interpretations regarding the same nationalist issue. As a result, the nuanced roles among various actors in co-constructing online nationalist discourse remain underexplored. This gap reflects a broader theoretical challenge in studies of digital nationalism, which often reduce platform-mediated public opinion to either elite opinion leaders or mobilized grassroots actors. By treating nationalist discourse as fragmented and relational, rather than actor-specific, this study aligns with emerging perspectives that view nationalism as co-constructed through platform dynamics and cross-group interaction.

In this paper, we will analyze how nationalist and rational discourses surrounding Fukushima wastewater release evolve by applying Structural Topic Modeling (STM). In doing so, we will provide dynamic and more nuanced analysis on how grassroots and KOLs co-construct and navigate between nationalistic and rational discourses. This perspective moves beyond the binary frameworks of “state-centric” or purely “bottom-up” assumption, seeking to offer comprehensive insights into how nationalist sentiments are strategically activated between two actors across different stages. Through this investigation, we expect to contribute to the understanding of latent trigger mechanisms that amplify nationalist discourse, particularly in relation to how victimhood serves as a reservoir for contemporary nationalist expression in Chinese social media. This approach situates the analysis within established frameworks of grievance nationalism and affective or victimized nationalism, which emphasize how historical injury and perceived injustice are recurrently mobilized to sustain national solidarity and moral superiority. In Chinese social media, environmental controversies such as the Fukushima wastewater release become readily nationalized as technical risks are reframed as symbolic threats to collective dignity, public health, and historical memory. Platform-mediated interactions enable both KOLs and grassroots users to translate environmental anxiety into affective narratives of victimhood and blame, thereby activating nationalism without explicit state orchestration. As a result, environmental communication functions as a politically legitimate conduit through which grievance-based nationalism is continuously reproduced and amplified online.

## Literature review and research question

### Reevaluate Chinese online political discourse: Nationalism VS. rationalism

The digital transformation of political discourse has fundamentally altered the conditions under which public discussion occurs, intensifying a core tension between rational deliberation and emotional mobilization. Habermas [27]’s foundational conception of the public sphere as a domain of rational-critical debate encounters new complexities in digital environments, where algorithmic amplification tends to privilege emotionally charged, out-group hostile content over nuanced argumentation [28]. This tension between rational and emotional discourse is not unique to deliberate democracy contexts—even in China’s authoritarian system, scholarship remains divided on whether nationalism serves as the dominant ideological force or whether digital platforms create space for more pluralistic political discourse.

Several strands of scholarship on digital nationalism converge in showing how Chinese online spaces, especially in the context of Sino–Japan relations, systematically transform historical grievance into contemporary nationalist mobilization through platform dynamics. Schneider’s study of the Nanjing Massacre shows how state control over digital infrastructure and algorithmic curation produces a highly homogeneous nationalist narrative, in which historical memory is continuously reactivated and stabilized across websites, encyclopedias, and hyperlink networks [29]. Building on this infrastructural account, Ng and Han demonstrate how everyday platform interactions further intensify this dynamic: in responses to the Japanese celebrity Sora Aoi on Weibo, nationalist antagonism toward Japan becomes intertwined with misogynistic abuse, revealing how affective and victimized nationalism is enacted through personalized and emotionally charged discourse rather than formal political argument [30]. Together, these findings resonate with Guo, Cheong, and Chen’s notion of “latent nationalism,” in which platform architectures and attention economies allow nationalist sentiment to be triggered with minimal deliberation [31]. Taken as a whole, this literature helps explain how environmental controversies involving Japan become rapidly nationalized in Chinese digital spaces, as technical risks are framed into historical injury and amplified through affective, algorithmically mediated public engagement.

Conversely, a competing body of scholarship challenges this nationalism-dominant narrative, arguing instead that Chinese digital spaces foster more pluralistic political discussions than is often assumed. Liu [32]’s qualitative analysis of online debates during the 2005 anti-Japan protests revealed that cyber nationalism functions not merely as an extension of state propaganda, but as a counterforce that creates space for negotiating national identity beyond official narratives. Although extreme nationalism exists, the online environment allows for debate and dissent that complicate simple state-society binaries. This finding echoed with Zhang [25]’s quantitative analysis, which demonstrated that Chinese online political discourse was not dominated by nationalistic views, but rather by pro-democracy perspectives that explicitly criticize government policies. These divergent findings indicate that existing scholarship may benefit from moving beyond the binary question of whether nationalism or pluralism dominates Chinese digital discourse. A more nuanced approach would examine how nationalist and rationalist modes of discourse interact and compete within the same digital spaces.

The Fukushima wastewater release provides an ideal case for examining how nationalist and rationalist discourse compete in Chinese digital spaces. This issue combines technical scientific questions about radiation safety and cosmopolitan concerns over global environment, alongside deep nationalist sensitivities regarding Japan’s historical role in China. Drawing on Habermas [27]’s concept of communicative rationality, which emphasizes reasoned argumentation aimed at mutual understanding, in this study we define rationalism as a mode of discourse grounded in evidentiary deliberation—scrutinizing claims through scientific data and logical coherence—and transnational norms, appealing to ethical frameworks that extend beyond parochial nationalism.

Our study aims to reveal how internet users navigate between nationalistic responses and rational discussions. Thus, the following research question is posited:

***RQ1: How does the relative prevalence of nationalism versus rationalism discourse shift throughout different phases of the Fukushima wastewater release?***

### Shaping cyber nationalism: Key opinion leaders (KOLs) VS. grassroots

The Fukushima wastewater release sparked both nationalist discussions and rational responses. While rational discourse offers an evidence-based perspective, nationalistic discourse is assumed to dominate. In recent years, Chinese online space has witnessed the marginalization of rational and moderate voices, a structural trend shaped by rigorous institutional regulations [33], algorithmic amplification of affective content [13], and sophisticated propaganda which is constantly adapting to new digital affordances [34–36]. Given this structural landscape, our study, while acknowledging the presence of rational discourse, focuses primarily on the dynamics of nationalist discourse.

If the rise of the internet initially led to a weakening of state control over public expressions of national belonging and “turned the hitherto state-centered logic of nationalism on its head” [37], the growing monopoly of platforms has enabled the state to reassert control over national imagination, while also opening doors for other political and corporate actors to interfere in the process [13]. This transformation has empowered multiple actors—state institutions, Key Opinion Leaders (KOLs), and grassroots users to shape nationalistic discourse within an increasingly complex digital ecosystem.

State-led nationalism has received considerable attention among scholars. Gries [38] argued that regime legitimacy is the key mediator accounting for the association and sequencing between popular nationalism and China’s Japan policy. Cairns [39] uncovered how government censorship on Weibo fluctuated throughout the Diaoyu/Senkaku dispute, reflecting a strategic response to manage nationalistic sentiment and prevent social instability. State-controlled nationalism exerts significant influence by shaping the information infrastructure through “techno-nationalism” and propaganda [40,41]. However, the direct mechanisms of state intervention often remain opaque due to undisclosed decision-making in authoritarian nations, making it difficult to measure their precise impact. Therefore, this study treats official nationalism as a structural backdrop, instead focusing on the observable interactions between KOLs and grassroots users.

Within the Chinese social media ecosystem, key opinion leaders (KOLs) serve as influential intermediaries in shaping public opinion. Weibo endorses these KOLs through verification badges, marking them as “Big Vs” with substantial follower bases and social influence [42]. Unlike paid subscription models on platforms such as X (formerly Twitter), Weibo enforces a real-name authentication system where verification is contingent upon professional credentials and quantitative influence metrics, including specific requirements for follower counts and readership. These verified accounts mainly fall into three categories: official media outlets representing party-state interests with diplomatic tones [43]; commercial “We-Media” (Zimeiti) accounts that align ideological correctness with “ideotainment”—a blend of propaganda and popular culture to maximize traffic [44,45]; and public intellectuals—such as former Global Times editor Hu Xijin—who offer nuanced policy interpretations while adhering to party-state interests [42]. Their verified status and large followings make them effective channels for top-down communication of state-sanctioned narratives. This influence was evident in Guo [46]’s analysis of the 2014 Hong Kong Umbrella Movement discussions, where mainland Chinese netizens echoed these opinion leaders’ paternalistic attitudes and nationalistic rhetoric, framing the protests as an anti-China conspiracy with Western backing.

Meanwhile, grassroots users have demonstrated an increasing agency in shaping nationalist narratives, although the nature of this mobilization has undergone a critical transition. Early studies highlighted the bottom-up dimensions of nationalistic expression, which were typically precipitated by specific geopolitical crises [47]. An example is the 2016 Diba Expedition to Facebook, where Taiwan presidential election triggered a voluntary online campaign through popular forms of Chinese memes [48]. However, recent scholarship suggests that this agency has evolved from reacting to clear political disputes to a hyper-sensitive vigilance known as “Ruhua” (辱华) or “insulting China” accusations. Wu and Zhang [49] argue that recent popular nationalism has shifted to a rigid defense of state dignity, which can be incited by seemingly innocuous content—such as a casual photo or a sports itinerary—provided it can be interpreted as challenging the state’s territorial integrity or historical narratives. This trend indicates that threshold for “insulting China” is lowered, allowing unrelated cultural slights to rapidly escalate into crises of national dignity.

Despite these valuable insights, the nuanced roles among various actors in co-constructing online nationalist discourse remain underexplored. Most studies focus on either top-down state influence or bottom-up grassroots participation, but rarely examine how different social actors construct and potentially diverge in their nationalist interpretations within the same discursive space. While verified KOLs typically amplify state narratives, grassroots users represent bottom-up participation in nationalist discourse. This study addresses this gap by examining the dynamic interplay between these two actors in constructing nationalist narratives, using the Fukushima wastewater release as a case study, leading to the following question:

***RQ2: What are the relative roles of grassroots users and KOLs in shaping nationalist narratives during the Fukushima event?***

## Data and methods

### Data collection and preprocessing

Data collection utilized the open-source GitHub tool dataabc/weibo-search, retrieving posts through two keywords: nuclear wastewater (核废水) and nuclear polluted water (核污水), commonly used in Chinese discourse and official reports. Original posts (原创微博) were crawled from July 4, 2023, to March 18, 2024, encompassing key events such as the IAEA’s assessment of Japan’s ocean discharge plan on July 4, 2023, Prime Minister Fumio Kishida’s announcement on August 22, 2023, and the completion of the fourth discharge round on March 17, 2024. A total of 70,759 original posts were collected, along with metadata including hashtags, verification status, and postdate.

Although each collected post contained at least one of the target keywords, a subset of the data contained noise that did not reflect genuine user opinions on Fukushima wastewater release. We manually excluded these non-substantive posts, which included: reposts from third-party platforms (e.g., Douyin, Little Red Book); lottery notifications (identified by the token sequence “Lottery Details”); posts consisting solely of hashtags; and unrelated topics ranging from entertainment news and investment advice to civil service exam tips that utilized Fukushima-related hashtags to increase visibility, a practice known as “hashtag hijacking.” While we acknowledge that keyword-based filtering has its limitations, efforts were made to filter out posts that did not genuinely reflect user opinions on the Fukushima wastewater issue. After filtering, 56,797 posts remained. Table 1 presents the distribution of Weibo posts after filtering by user type, showing that ordinary accounts contributed the majority of posts (71.16%, n = 40,416) compared to verified accounts (28.84%, n = 16,381) out of a total of 56,797 posts. Table 2 shows the top 10 Chinese hashtags found in posts after filtering that matched the keyword search, along with their English translations, drawn from a total of 12,732 instances of hashtags.

**Table 1:** Distribution of Weibo Posts by User Type.

User Type	Number of Weibo Posts	Percentage (%)
Ordinary Account	40,416	71.16
Verified Account	16,381	28.84
**Total**	56,797	100.00

*Note*: The table summarizes the distribution of Weibo posts by user type, showing both the absolute numbers and percentages.

**Table 2:** Top 10 Most Frequent Hashtags Extracted from Collected Weibo (*n* = 12,732).

Hashtag	English Translation	Count	Percentage (%)
日本核废水	Japan’s nuclear wastewater	5,482	8.63
日本政府正式决定福岛核废水排海	Japanese government officially decides to discharge Fukushima nuclear wastewater into the sea	2,479	3.90
日本核污水	Japan’s nuclear contaminated water	1,987	3.13
坚决抵制日本排放核污水	Resolutely oppose Japan’s discharge of nuclear contaminated water	1,307	2.06
日本核污水57天将污染半个太平洋	Japan’s nuclear contaminated water will pollute half of the Pacific Ocean in 57 days	1,193	1.88
核污水	Nuclear contaminated water	1,174	1.85
福岛核污水泄漏系因忘关手动阀门	Fukushima nuclear contaminated water leak due to forgetting to close manual valve	1,028	1.62
日本	Japan	891	1.40
研究称日本核污水排海240天到达中国	Study claims Japan’s nuclear contaminated water will reach China in 240 days after discharge	778	1.22
核废水	Nuclear wastewater	668	1.05

*Note*: The table summarizes the top 10 hashtags related to the Fukushima wastewater release, showing counts and percentages.

We subsequently cleaned the text by removing structural noise, including username mentions, Weibo video tags, URLs, and “Super Topics” (超话). Super Topics are interest-based community tags on Weibo, mainly used for celebrity fandoms and gaming. As these tags represent platform-specific metadata rather than substantive discourse regarding the wastewater incident, they were stripped to prevent semantic noises. Finally, stopwords were removed and text segmentation was performed using the Jieba package designed for Chinese text. To ensure the topic model captured distinct thematic variations rather than the common denominator of the dataset, the search keywords themselves (“nuclear wastewater” and “nuclear contaminated water”) were treated as corpus-specific stop words and removed during tokenization.

### Measuring dynamic discourse with structural topic modeling (STM)

In this study, Structural Topic Model (STM) was chosen due to its unique advantages. One of the primary benefits of employing STM is its ability to incorporate document-level metadata (covariates) to influence both topic prevalence and content [50]. This feature distinguishes STM from traditional topic modeling methods such as LDA, which typically utilize Gibbs sampling or variational inference for estimating model parameters and focus solely on word distributions [51].

We aimed to examine the evolution of discourses as the discharge process progresses and differential issue attention between key opinion leaders and grassroots, the date of the posts and user type emerged as crucial covariates. To analyze the relationship between our chosen covariates and topic prevalence, we employed a regression-based approach within the STM framework. The date of the posts was modeled using a smooth function spline to capture potentially non-linear temporal effects on topic prevalence, allowing for flexible modeling of time-varying effects without imposing a rigid linear structure [52]. User type, categorized as key opinion leaders (KOLs) or grassroots, was included as a categorical covariate. We employed a global approach to uncertainty quantification, ensuring robust and conservative estimates [53], and estimated the individual and interaction effect of covariates separately.

To prepare the textual data for STM, we utilized the textProcessor function. A critical parameter in this step was the determination of token length. To align with the linguistic characteristics of Chinese, where unigrams are often functional or ambiguous, and substantive meaning is encoded in compound words, we filtered out all tokens consisting of a single character, ensuring that the model was constructed exclusively on multi-character tokens. It resulted in the removal of 271 documents that no longer contained valid text tokens, yielding the final analytical corpus of 56,526 documents reported in our results.

**Fig 1:**
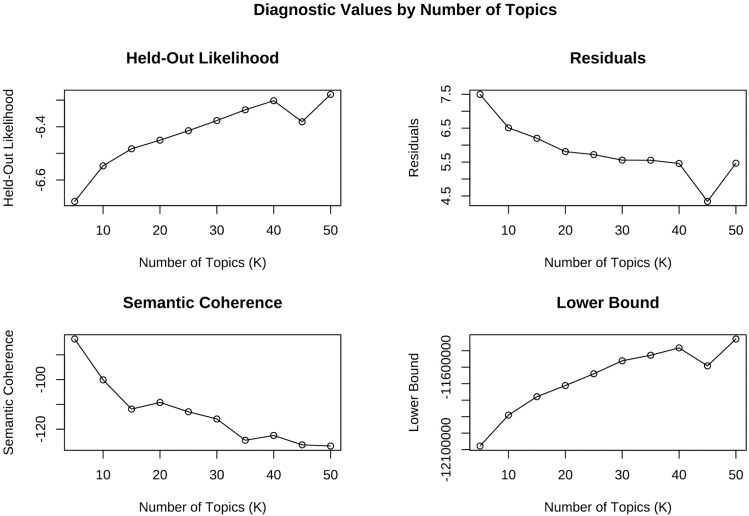
Wide-Range Number of Topics Screening (K = 5–50). Structural topic model diagnostics (held-out likelihood, semantic coherence, residuals, and variational lower bound) surveyed over a wide topic range (K = 5–50) to enable broad exploration and identification of a plausible region for the optimal number of topics.

**Fig 2:**
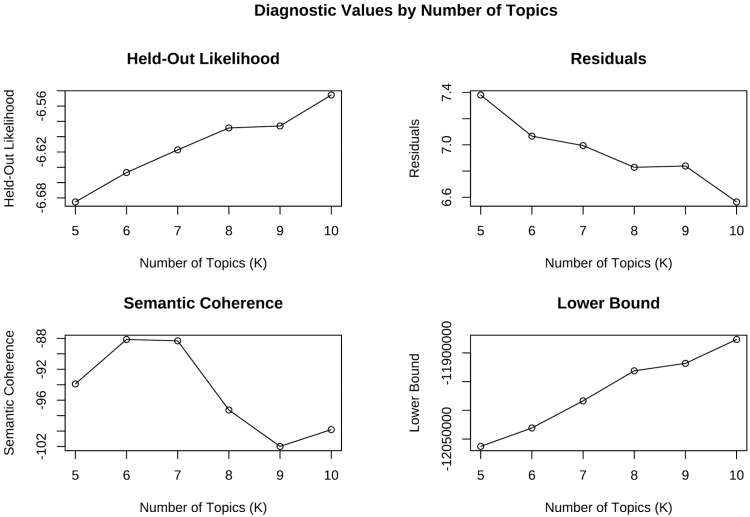
Narrow-Range Number of Topics Selection (K = 5–10). Structural topic model diagnostics (held-out likelihood, semantic coherence, residuals, and variational lower bound) evaluated over a deliberately narrow topic range (K = 5–10) to enable fine-grained, precise selection of the optimal number of topics.

As STM is an unsupervised model, another crucial initial step is determining the optimal number of topics to represent the text corpus. To achieve this, searchK function was utilized to compute diagnostic values for models with different values of K. Our topic number selection followed a two-step validation process to balance statistical rigor and interpretability, leading to the choice of 7 as the optimal number of topics due to lower residuals and higher semantic coherence. Firstly, we conducted a wide-range screening (K = 5–50) as Fig 1 shows, where statistical fit metrics showed only marginal improvements as the number of topics increased. The stabilization of the lower bound beyond K = 10 indicated diminishing returns [54]. Then, we performed a narrow-range selection (K = 5–10) as Fig 2 shows. The Ideal solutions are characterized by fewer residuals and higher exclusivity, variational lower bound, and semantic coherence [55]. Our aim was to derive actionable insights rather than corpus-specific optimization, making semantic coherence the most crucial metric due to its strong correlation with human judgment (ρ=0.78) and its precedence over likelihood in applied settings [56]. K = 7 was selected as the optimal number of topics because it demonstrated favorable semantic coherence (−88.31) while maintaining low residuals (6.99). Models with *K* > 7 exhibited a substantial deterioration in semantic coherence, with K = 8 dropping to −97.27 and K = 10 plummeting to −99.81—representing a 10% and 13% decrease respectively from K = 7. Although K = 10 achieved slightly lower residuals (6.56), this marginal improvement was overshadowed by the dramatic loss in semantic interpretability, making K = 7 the optimal choice that balances model fit with meaningful topic coherence.

## Results

### Two antagonisms in nationalistic discourse: Anti-Japanese and Anti-Western

**Fig 3:**
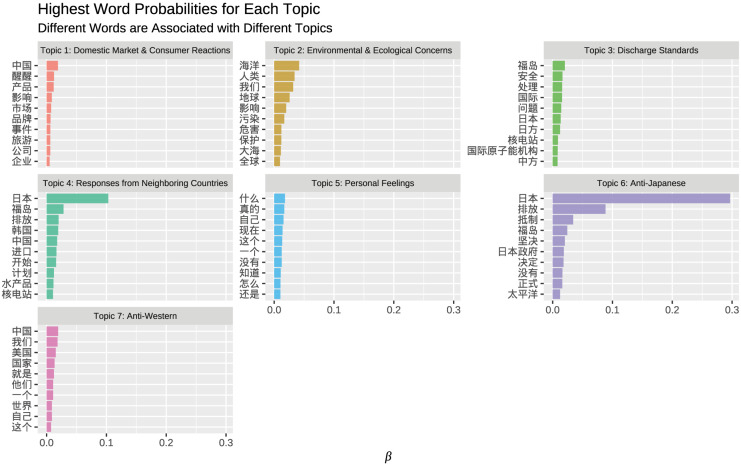
Highest Word Probability for Each Topic. Top-probability words for each topic in the 7-topic structural topic model of Chinese texts. Bars represent highest estimated token probabilities (β).

**Fig 4:**
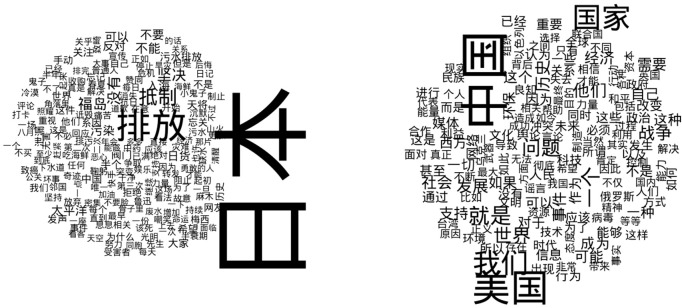
Nationalism Discourse Wordcloud. Chinese-language word cloud of Anti-Japanese (Topic 6) and Anti-Western (Topic 7) Weibo posts. Token size is proportional to relative frequency after basic preprocessing. High-frequency tokens concentrate on: National actors and identity (中国 China, 日本 Japan, 民族 nation, 我们 we, 美国 USA) and emotive geopolitical critique (抵制 boycott, 鬼子 derogatory anti‑Japanese term, 战争 war).

To analyze the discourse patterns in the dataset, we employed a mixed-method approach combining computational topic modeling and qualitative coding. We generated a Structural Topic Model (STM) from 56,526 documents using a dictionary of 4,461 terms, resulting in 7 distinct topics (Fig 3). Manual coding categorized these topics based on nationalist and rationalist discourses. Nationalist discourse, marked by anti-Japanese and anti-Western sentiments (Table 3), appeared in Topics 6 (Anti-Japanese) and 7 (Anti-Western). In contrast, rationalist discourse, characterized by evidence-based arguments and global humanitarian concerns, was found in Topics 2 (Environmental & Ecological Concerns) and 3 (Discharge Standards). Topics 1, 4, and 5 were classified as ‘other/mixture,’ covering market impacts, regional responses, and sparse personal reactions. This hierarchical analysis improved interpretability and facilitated theoretical saturation evaluation [57]. Fig 4 presents a word cloud of terms associated with nationalistic topics (Topics 6 & 7).

**Table 3:** Manual Categorization Based on Structural Topic Modeling Result.

Topic	Topic Name	Representative Tokens	Category
1	Domestic Market & Consumer Reactions	Seafood, Tourism, Product, Essence, Impact, China, Market	Other/Mixture
2	Environmental & Ecological Concerns	Earth, Marine life, Mankind, Destruction, Nature, Environmental Protection, Save	Rationalism
3	Discharge Standards	IAEA, Radioactive material, Assessment, Safety, Endorsement, Pure Water, Cooling Water	Rationalism
4	Responses from Neighboring Countries	South Korea, Import, Seafood, Fumio Kishida, Hunger Strike, Prohibition, Sardine	Other/Mixture
5	Personal Feelings	Feel, Then, Think, Like, Unwilling to, Seems like, But	Other/Mixture
6	Anti-Japanese	Firmly, Boycott, Japanese Products, Marco Polo Bridge, Manual, Forget to turn off, Contamination	Nationalism
7	Anti-Western	The West, Science and Technology, Civilization, Conscience, Israel, Justice, USA	Nationalism

**Notes:** This table categorizes topics based on results from structural topic modeling. Each topic is manually labeled and grouped into categories for further analysis.

The first layer of antagonism shows how historical grievances serve as a foundation 331 for anti-Japanese sentiment:

#Japan’s nuclear wastewater# I suggest that our country (China) should report more related news. This thing is much scarier than COVID-19 and its impact on humanity will last for generations. The Japanese appear polite on the surface, but in private, they are still the same Japanese devils. The atrocities of Unit 731 have not been brought to justice, and now there is nuclear wastewater. Everyone should condemn Japan’s discharge, for the sake of the Earth, for all living beings on Earth, and for all humanity.

In Topic 6, the antagonistic logic primarily draws upon historical trauma, particularly referencing World War II atrocities (“Japanese devils” and “Unit 731”). This discourse strategically juxtaposes Japan’s contemporary actions with historical grievances, suggesting a continuity of malevolent intent beneath a veneer of civility (“appear polite on the surface”). In this context, the controversial global environmental issue is reframed as symbolic of Japan’s historical disregard for global well-being—an act rooted in the same moral failings that characterized its wartime atrocities. This narrative also implies a victimhood mentality by portraying China as a moral guardian standing against Japan’s recurring harm to humanity.

The second layer indicates a conspiracy-oriented antagonism that extends beyond bilateral tensions to implicate the broader Western power structure:

Tokyo Electric Power Company in Japan is actually an American company! I’ve always wondered why Japan insists on discharging nuclear wastewater into the ocean, which harms others without benefiting itself. Meanwhile, the United States and Europe, who usually champion environmental protection, have collectively remained silent, and even the International Atomic Energy Agency has been bought off. Just look at the major shareholders of Tokyo Electric Power Company! The largest shareholder of Tokyo Electric Power Company is the renowned Wall Street giant BlackRock, which is also a major financier of the Ukrainian comedian. The second-largest shareholder, Vanguard, is also a Wall Street giant, both are Jewish capital……

In Topic 7, the conspiracy theories about Western financial institutions reflect how external threats are perceived as coordinated actions by Western powers. The terms “Jewish capital” and “US-Japan Water” reflect an antagonistic worldview among Chinese internet users that frames global politics through a US-China antagonistic lens, categorizing the international community into two camps: ‘China and its allies’ and ‘the US and its allies.’ Within this framework, the IAEA’s authorization is interpreted as a manifestation of how the US-Japan alliance operates to compromise China’s national interests, implying a sense of collective victimhood which positions China as a target of systemic Western collusion.

Overall, the STM identifies two dominant nationalist antagonisms—anti-Japanese (Topic 6) and anti-Western (Topic 7)—that structure how the Fukushima wastewater issue is discussed online. At the evaluative level, Topic 6 frames Japan’s discharge through historical grievance and moral condemnation, linking contemporary environmental risk to wartime trauma, while Topic 7 interprets the issue through conspiracy-oriented geopolitical narratives that position Western actors as coordinated threats to China. Together, these topics reframe a technical environmental controversy as a nationalist narrative centered on collective victimhood and moral opposition, demonstrating how environmental discourse is systematically absorbed into broader patterns of digital nationalist antagonism.

### Dynamics of nationalism and rationalism discourses across key phases

**Fig 5:**
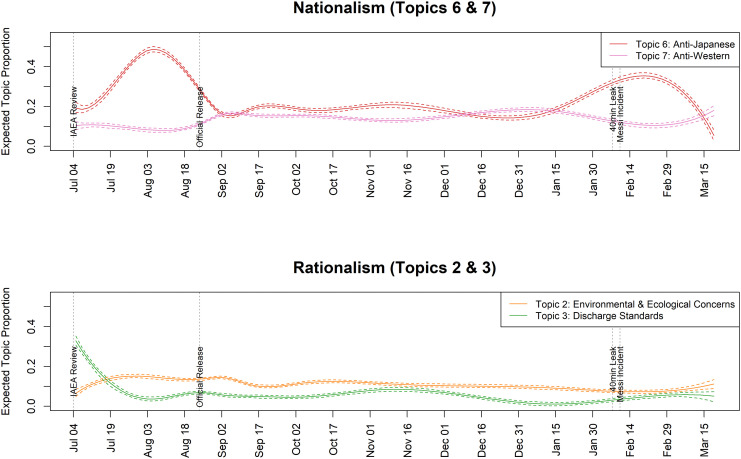
Topic Proportion Trends Over Time. It shows how topic proportions between nationalism and rationalism fluctuate throughout the observed period. The solid lines represent the expected proportions of each topic, while the dotted lines denote the 95% confidence intervals. Visually, periods where confidence intervals do not overlap suggest separation in the prevalence of the respective topics. Conversely, overlapping confidence intervals suggest insufficient evidence to assert a visual separation. Formal inference relies on the model-based estimates reported in the text.

Fig 5 visualizes the time-varying proportions of nationalism and rationalism topic categories.

### Phase 1: The Intensification of Nationalism (Early July 2023 – Early August 2023)

The first phase illustrates a dynamic interplay between nationalism and rationalism, with rational discourse (Topics 2 and 3) exceeding nationalist topics (Topics 6 and 7) during the critical window of July 4–11, 2023, coinciding with the IAEA’s safety review release. Rationalism initially dominated, peaking at a combined proportion of 0.41 on July 4. Statistical divergence occurred between July 11 and July 14; the proportion of Topic 3 declined sharply, while Topic 6 exhibited an inflection point, rising from 0.18 to 0.45. By July 14, the 95% Confidence Intervals (CIs) of Topic 6 [0.207, 0.225] and Topic 3 [0.145, 0.162] ceased to overlap, confirming a statistically significant transition (*p* < .05) toward nationalist framing. This shift aligns with the immediate aftermath of the IAEA’s safety review, suggesting that technical assurances acted as a brief stimulus for rational debate before being eclipsed by grassroots nationalist mobilization.

### Phase 2: Stabilization and Diffusion (Mid-August 2023 – January 2024)

Seven days right before Japan’s official discharge commencement (August 24, 2023), rational concerns resurged briefly, with Topic 2 (Environmental & Ecological) peaking at 0.192 (95% CI [0.189, 0.196]) on August 17—a last gasp of technical discourse before nationalist narratives solidified. Nationalism retained structural dominance compared with rationalism, as the combined proportion of Topic 6 and Topic 7 is larger than that of Topic 2 and Topic 3 across this phase.

### Phase 3: Resurgence of Nationalism (February 2024)

A dramatic nationalist resurgence occurred in February 2024, with Topic 6 (Anti-Japanese) surging to 0.379 (95% CI [0.373, 0.386]) on Feb 18—its highest level since August 2023. This was triggered by two events: A technical mishap (40-minute Fukushima leak on February 7), reigniting distrust in Japan’s stewardship, and the “Messi incident” (February 10), where the athlete’s perceived snub of Hong Kong in favor of Japan amplified anti-Japanese sentiment.

Fukushima nuclear wastewater leak is due to forgetting to close the manual valve. It’s not forgetting to close the manual valve, it’s forgetting the basics of being human. Japan is doing things that are not humane!

#Why Did Messi Only Respond to Missing The Game After Arriving In Japan? # Because of the small island’s nuclear waste treatment.

In Chinese cyberspace, the actions of foreign celebrities are frequently viewed through a nationalistic lens, where gestures can be interpreted as signs of either respect or disrespect toward China. The controversy surrounding Lionel Messi exemplifies this dynamic: his absence from a Hong Kong match and subsequent visit to Japan sparked widespread accusations of “insulting China” (辱华), with internet users criticizing his apparent preference for Japan. This public reaction suggests that strong national pride may transform perceived slights into feelings of victimization. Despite China’s growing global influence, online discourses appear to remain highly sensitive to actions that are perceived as prioritizing other nations – particularly Japan – over China. This sensitivity manifests in collective indignation, often expressed through satirical comments, as seen when netizens linked Messi’s behavior to Japan’s controversial nuclear wastewater discharge. Such responses arguably reflect a deeper underlying narrative. As China achieves greater economic and cultural milestones, there is an expectation of corresponding international recognition. This external validation is crucial for putting the “Century of National Humiliation” (百年国耻) firmly in the past. Therefore, any perceived disrespect challenges this modern narrative of national rejuvenation. It may easily serve to reawaken deep-seated historical feelings of victimization.

These findings complicate the notion that Chinese social media discourse is uniformly nationalistic. While nationalist narratives ultimately dominated discussions of Japan’s Fukushima wastewater release, the initial phase (July 4–11, 2023) revealed a coexistence of rationalism and nationalist perspectives. During this critical window, technical concerns about discharge standards (Topic 3) and environmental impacts (Topic 2) competed vigorously with anti-Japanese (Topic 6) and anti-Western (Topic 7). But the balance proved fragile as nationalistic discourse, especially anti-Japanese (Topic 6) became mainstream. Notably, anti-Western sentiment (Topic 7) maintained a stable presence (0.06–0.16 proportions) even as anti-Japanese rhetoric fluctuated wildly. This stability may suggest that anti-Western narrative operate as a stable structural feature of Chinese digital nationalism, whereas anti-Japanese sentiment functions as a situational amplifier activated by specific triggers. The latter phenomenon—where a sports figure’s itinerary reignited historical animosities—exemplifies what we term latent nationalist sensitivity: a psychological state where perceived offense to China rapidly reactivates historical victimhood, transforming trivial incidents into crises of national dignity. This mechanism, while resonating with earlier findings of latent nationalism [31], exemplifies a unique characteristic of Chinese digital nationalism, where “insulting China”(辱华) narratives are rapidly amplified through online discourse.

### The rise of grassroots nationalism in Sino-Japan conflict

**Fig 6:**
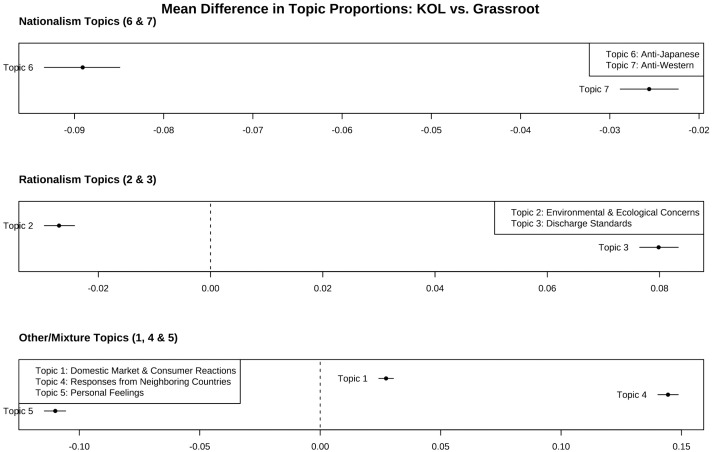
Estimated Effect of User Type on Topics. The x-axis represents the difference in topic prevalence, where positive values indicate higher prevalence among KOLs and negative values indicate higher prevalence among grassroots. Each point on the plot represents the estimated mean difference for a topic, with horizontal lines showing the 95% confidence intervals; if a confidence interval does not cross zero, it suggests a statistically significant difference in topic prevalence between the two user types for that topic.

Fig 6 shows the static estimated mean difference in topic proportion between KOLs and grassroots, where the difference is calculated as (verified account – unverified account).

Raw, volatile nationalism targeting Japan was primarily a bottom-up phenomenon as grassroots users significantly dominated the Topic 6 (Anti-Japanese) discourse (Effect Size = −0.089, 95% CI [−0.093, −0.085]). While grassroots users also led the discussion on Topic 7 (Anti-Western), the gap between the two groups was narrower (Effect Size = −0.026, 95% CI [−0.029, −0.022]). This suggests that although KOLs largely avoided direct anti-Japanese antagonism, they were more aligned with the general public regarding broader anti-Western geopolitical framing. In contrast, KOLs functioned as anchors for diplomatic and rational discourse, showing the most substantial positive preference for Topic 4 (Responses from Neighboring Countries) (Effect Size = 0.144, 95% CI [0.140, 0.149]) and Topic 3 (Discharge Standards) (Effect Size = 0.080, 95% CI [0.077, 0.083]).

**Fig 7:**
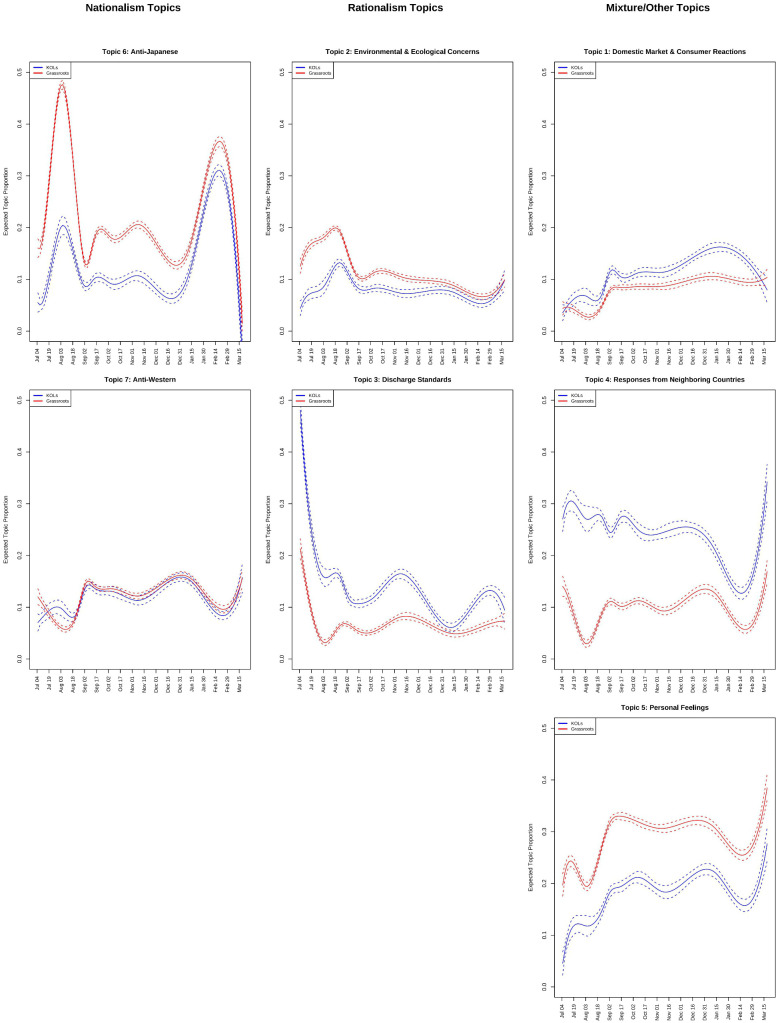
Temporal analyses on topic proportions across KOLs and grassroots. The figure provides temporal expected topic proportions grouped into three categories(nationalism, rationalism and other/mixture) between KOLs and grassroots. Blue denotes KOL; red denotes grassroots accounts. Solid lines show posterior mean expected topic proportions; dotted lines trace the upper and lower 95% confidence interval bounds.

Fig 7 shows a more detailed and time-specific view, revealing that these differences were not consistent across time. Notably, the relationship between grassroots and KOLs is not a simple binary of top-down orchestration versus bottom-up expression, but rather depends on the specific target of nationalism.

For anti-Japanese discourse (Topic 6), the divergence between grassroots and KOLs remained robust throughout the entire observation period. Grassroots users took a significantly higher proportion of attention to this topic than KOLs, with the difference remaining statistically significant at all time points. Even at the point of closest convergence on February 5, 2024, a clear gap persisted: grassroots mean was 0.295 (95% CI: [0.287, 0.302]) compared to the KOL mean of 0.265 (95% CI: [0.254, 0.276]). This indicates that while the intensity of the gap fluctuated, KOLs constantly maintained a lower profile toward direct anti-Japanese rhetoric compared to grassroots users.

However, with anti-Western discourse (Topic 7), the gap vanished during initial stage of discharge. Specifically, from July 17 to August 14, 2023, the difference in expected topic proportions became statistically insignificant. Within this window, particularly on July 30, KOLs exhibited a higher mean proportion (0.088, 95% CI: [0.074, 0.101]) than grassroots users (0.082, 95% CI: [0.077, 0.088]). Unlike the persistent stratification seen in anti-Japanese discourse, anti-Western discourse saw a temporary synchronization where state-affiliated attention aligned with grassroots sentiment during certain period.

These findings complicate the binary paradigm of Chinese digital nationalism as either purely state-orchestrated or entirely bottom-up, pointing instead to a dynamic and nuanced interplay between the state and populace. While grassroots users drove the volatile anti-Japanese sentiment (Topic 6), the relative silence of Key Opinion Leaders (KOLs) may suggest a degree of strategic restraint, permitting organic expression without explicit official endorsement. However, the discursive synchronization observed in anti-Western discourse (Topic 7) suggests that state-aligned KOLs may opportunistically leverage the public’s victimhood-centric worldview at certain junctures. Together, these patterns indicate that rather than uniformly dictating nationalist expression, the state apparatus may effectively modulate it by selectively channeling grassroots sentiment to support broader geopolitical objectives, while preserving diplomatic flexibility.

## Conclusion and discussion

Through the examination of online discourses surrounding the Fukushima wastewater release, this study seeks to contribute to existing understanding of digital nationalism in two ways. First, by employing Structural Topic Modeling (STM), the analysis reveals that although rational discourse emphasizing technical and environmental concerns coexisted and competed with nationalist narratives briefly at the initial stage, the discourse rapidly transformed into predominantly nationalist rhetoric driven by grassroots users. Nationalism discourse was framed through two antagonisms: 1) direct, volatile anti-Japanese sentiment rooted in historical grievances and 2) broader, consistent anti-Western skepticism anchored in geopolitical rivalries. Notably, the study identified latent nationalist sensitivity as a recent pattern in Chinese cyberspace: marked by a victimhood-oriented interpretation of international events, nationalism proved highly reactive to seemingly unrelated triggers, such as perceived insults to China, where anger toward Japan and concerns about China’s national dignity combined to reignite anti-Japanese sentiment. This finding advances our understanding of how nationalist sentiments operate in everyday digital spaces, moving beyond the traditional focus on orchestrated national events [58].

Second, this study provides empirical evidence that complicates both purely bottom-up and top-down assumptions about Chinese cyber nationalism by measuring the dynamic co-construction of nationalistic discourses between Key Opinion Leaders (KOLs) and grassroots users. Previous research uncovered KOLs’ agenda-setting role in anti-US and anti-West narratives [59], but our findings revealed a more nuanced interplay. While KOLs tended to focus on the technical aspects of contentious issues, grassroots users dominated nationalistic narratives, particularly those expressing anti-Japanese sentiment. Interestingly, during certain stages, anti-Western discourse appeared more prominent among KOLs than grassroots users. This finding contributes to broader debates on elite-grassroots dynamics in digital authoritarianism by mapping how emotional mobilization and strategic stability are negotiated in real-time. It is worth noting that the tension observed here between elite technocracy and grassroots affective mobilization transcends political boundaries. This dynamic illustrates global discursive trends in the digital age, as seen in the COVID-19 vaccine discussions, where evidence-based narratives from experts often struggled to overshadow grassroots discourses driven by identity, skepticism, and populist sentiments [60].

While previous studies have focused on youth-driven online nationalism through “soft” content like memes and fandom culture [48,61], this research revealed a broader and more aggressive pattern of nationalist expression. Examining a diverse demographic beyond the “Little Pink” (小粉红) youth segment, our findings implied that anti-Japanese discourse triggered markedly harsher and more aggressive rhetoric. This variation demonstrates that grassroots nationalism is not monolithic but rather manifests differently across user demographics and varies based on the specific target of nationalist sentiment, with Japan evoking intense responses compared to other external entities like Taiwan.

Earlier scholarship featured China’s internet as a “safety valve” for nationalist expression without street mobilization and concrete actions [62,63]. However, recent events blur the line between online discourse and real-world consequences. The September 18 attack on a Japanese schoolchild in Shenzhen [64] raises concerns about the offline implications of unchecked digital nationalism. Existing research indicates that inflammatory online rhetoric targeting specific groups often correlates with an increased probability of offline violence [65–67].

This study has certain limitations that must be acknowledged. First, the analysis relies exclusively on posts that remained visible on Weibo, introducing a specific form of survivorship bias akin to informative censoring [68]. Given the regulatory environment of Chinese internet, platform moderation system may remove content that deviates from the official stance, particularly pro-Japanese or highly critical rationalist discourse, while permitting indignant popular nationalists [69]. Consequently, the dataset likely reflects a curated reality rather than a complete picture of organic public opinion. Second, while verification status served as a proxy for Key Opinion Leaders (KOLs), this operationalization treats a heterogeneous group as a monolith. As noted, verified accounts encompass diverse actors, including state media, commercial influencers (“We-Media”), and independent intellectuals. Treating them as a single category may obscure the nuanced distinctions between state-directed propaganda and profit-driven clickbait nationalism. Future research could benefit from fine-grained sub-classification to disentangle these competing motivations. Finally, this study focuses on a single event on a single platform. Other digital spaces, such as Douyin or BiliBili, may exhibit different discursive dynamics. Future research may consider comparative analyses across multiple platforms and diversified contexts to provide a more comprehensive understanding of how nationalism manifests in the digital age.

## Supporting information

S1 Table Topic proportions and confidence intervals for Topics 2, 3, 5, 6, and 7 in Figure 5. (XLSX)

S2 Table Effect sizes and confidence intervals across all topics in Figure 6. (CSV)

S3 Table Topic proportions and confidence intervals for anti-Japanese discourse (Topic 6) in Figure 7. (CSV)

S4 Table Topic proportions and confidence intervals for anti-Western discourse (Topic 7) in Figure 7. (CSV)

S5 File Replication data and code. All data and code necessary to replicate the findings of this study are available in this file. The full dataset collected from Weibo for the STM analysis is provided in fukushima_data.csv in its original language. To facilitate verification for English readers, a subsample of 30 representative posts with English translations is provided in random_rows_translated.csv. All corresponding analysis code is provided in stm_script.R.
(ZIP)
